# Construction of cellulose-degrading microbial consortium and evaluation of their ability to degrade spent mushroom substrate

**DOI:** 10.3389/fmicb.2024.1356903

**Published:** 2024-03-14

**Authors:** Junqiao Long, Xueli Wang, Shuyi Qiu, Wei Zhou, Shaoqi Zhou, Kaiwei Shen, Lili Xie, Xiao Ma, Xuyi Zhang

**Affiliations:** ^1^Guizhou Province Key Laboratory of Fermentation Engineering and Biopharmacy, Guizhou University, Guiyang, China; ^2^College of Liquor and Food Engineering, Guizhou University, Guiyang, China; ^3^Guizhou Academy of Sciences, Guiyang, China; ^4^College of Resources and Environmental Engineering, Guizhou University, Guiyang, China

**Keywords:** cellulose-degrading strains, microbial consortium, cellulase, spent mushroom substrate, biomass degradation

## Abstract

**Introduction:**

Spent mushroom substrate (SMS) is a solid waste in agricultural production that contains abundant lignocellulosic fibers. The indiscriminate disposal of SMS will lead to significant resource waste and pollution of the surrounding environment.The isolation and screening of microorganisms with high cellulase degradation capacity is the key to improving SMS utilization.

**Methods:**

The cellulose-degrading microbial consortiums were constructed through antagonism and enzyme activity test. The effect of microbial consortiums on lignocellulose degradation was systematically evaluated by SMS liquid fermentation experiments.

**Results:**

In this study, four strains of cellulose-degrading bacteria were screened, and F16, F, and F7 were identified as *B. amyloliquefaciens*, PX1 identified as *B. velezensis*. At the same time, two groups of cellulose efficient degrading microbial consortiums (PX1 + F7 and F16 + F) were successfully constructed. When SMS was used as the sole carbon source, their carboxymethyl cellulase (CMCase) activities were 225.16 and 156.63 U/mL, respectively, and the filter paper enzyme (FPase) activities were 1.91 and 1.64 U/mL, respectively. PX1 + F7 had the highest degradation rate of hemicellulose and lignin, reaching 52.96% and 52.13%, respectively, and the degradation rate of F16 + F was as high as 56.30%. Field emission scanning electron microscopy (FESEM) analysis showed that the surface microstructure of SMS changed significantly after microbial consortiums treatment, and the change of absorption peak in Fourier transform infrared spectroscopy (FTIR) and the increase of crystallinity in X-ray diffraction (XRD) confirmed that the microbial consortiums had an actual degradation effect on SMS. The results showed that PX1 + F7 and F16 + F could effectively secrete cellulase and degrade cellulose, which had practical significance for the degradation of SMS.

**Discussion:**

In this study, the constructed PX1 + F7 and F16 + F strains can effectively secrete cellulase and degrade cellulose, which holds practical significance in the degradation of SMS. The results can provide technical support for treating high-cellulose solid waste and for the comprehensive utilization of biomass resources.

## 1 Introduction

China is a major producer of edible mushrooms, with an annual output of approximately 13.2 million tons, accounting for 75% of the total global production (Xie et al., 2019; Zhou et al., 2020). Spent mushroom substrate (SMS), a waste product in the production of edible mushrooms, is mainly composed of corn cobs, cottonseed shells, straw, sawdust, etc. (Zhu et al., 2012). Typically, for every 1 kg of edible mushrooms produced, about 5 kg of SMS is also generated as a by-product (Chang et al., 2021). According to this ratio, it is estimated that China’s annual production of SMS will exceed 2 × 10^4^ million tons (Lou et al., 2017). Currently, SMS is mainly treated by incineration or landfilling, which may cause various environmental problems, including soil and air pollution (Loganathan et al., 2023). Indeed, SMS contains abundant lignocellulosic fibers, as well as lignocellulose-degrading enzymes such as laccase (Lac) and manganese peroxidase (MnP), polysaccharides, proteins, organic acids, and other bioactive substances, and has high ecological utilization value (Branà et al., 2020; Gao et al., 2023).

Cellulose serves as a crucial component of SMS and stands as one of the primary sources of renewable biomass. It is also a limiting factor in the comprehensive utilization of SMS (Wang and Rong, 2022). Therefore, enhancing the conversion rate of cellulose is of utmost significance for the green and sustainable development of the edible mushroom industry. To date, cellulose conversion methods fall into three categories, which are involved in physical, chemical, and biological methods. Both physical methods (such as steam explosion and crushing) and chemical methods (such as using acids and alkalis) have problems, including high energy consumption and high costs. At the same time, chemical methods also lead to increased environmental pollution due to the production of chemical residues such as furan acids, phenols, and furans (Dar et al., 2022; Sai Bharadwaj et al., 2023; Zhao et al., 2023). In comparison, the biological method using cellulase has attracted the attention of researchers due to its environmental friendliness, mild conditions, and economic advantages (Andlar et al., 2018). Cellulase is an important complex enzyme system, which is composed of endoglucanases (C1), exoglucanases (Cex), cellulose-hydrolysing enzymes (CBH), and β-glucosidases (β-Gase) (de Souza and Kawaguti, 2021). During the hydrolysis process of cellulose, C1 acts on the amorphous region of cellulose, randomly breaking the β-1,4-glycosidic bonds within the molecules to form long-chain cellulose oligosaccharides. Cex or CBH then breaks down cellulose and cellulose oligosaccharides from the ends, releasing glucose and cellobiose (Sajith et al., 2016). Subsequently, cellobiose can be converted into glucose through the action of β-Gase (Garcia-Galindo et al., 2019). It has been found that microorganisms are an important source of cellulase. Hence, the isolation and screening of cellulose-degrading bacteria under different environmental conditions are of great significance for the of cellulose (Gao et al., 2017). Currently, strains with high cellulase production have been isolated and screened from animal feces (Dhakal et al., 2020), decaying wood (Haidar et al., 2021), and soil (Wang et al., 2023), including bacteria such as *Bacillus*, *Thermotogales*, and *Bacteroidales*, as well as fungi such as *Trichoderma harzianum*, *Trichoderma viride*, and *Penicillium* (Weimer, 2022).

The degradation of lignocellulosic fibers in nature is the result of the collective action of microbial communities. Different strains in microbial communities can drive metabolic specialization through mechanisms such as metabolic complementarity, enhancing the adaptability of the microbial community to various environments, and thus improving the degradation rate of lignocellulosic materials. Therefore, research has shown that the synergistic interaction between microbial communities can compensate for the deficiencies of single strains in degrading lignocellulosic fibers (Zhang et al., 2023). For example, Hu et al. (2017) found that the moderate addition of *Trichoderma reesei* can promote the interaction between microbial communities, resulting in higher levels of filter paper enzyme (FPase), Carboxymethyl Cellulase (CMCase), and xylanase enzyme activities, thereby improving overall cellulase activity. Chen et al. (2021) constructed a psychrophilic stalk-degrading bacterial consortium (PSBC), and used it to treat cornstalks for 50 days at 10–15°C. The degradation rate of cornstalk reached 59.3%, indicating that the PSBC consortium accelerated the decomposition of cornstalk. It can be seen that the construction of high-yield cellulase bacterial strains has a positive effect on improving the utilization efficiency of lignocellulosic materials.

The purpose of this article is to screen and isolate efficient cellulose-degrading bacteria from SMS, laying the foundation for the construction of cellulose-degrading microbial consortium. At the same time, by comprehensively analyzing and evaluating the degradation effect of microbial consortium on SMS through changes in the SMS hydrolysis weight loss rate, composition, and fiber structure characteristics. The findings provide technical support for the treatment of high-cellulose solid waste represented by SMS and the comprehensive utilization of biomass resources.

## 2 Materials and methods

### 2.1 Materials for experiments

The SMS was collected from Bijie city, Guizhou Province, China. The collected samples were divided into two parts: one part was used as the isolation material for cellulose-degrading bacteria, and the other part was used for pulverization using a multifunctional pulverizer (BL-F001, Baoli Scientific Research Apparatuses Co., LYD, Zhejiang, China). The samples were sieved through a 60-mesh sieve, and then dried at 90°C for more than 48 h. The SMS had a cellulose content of 21.87%, hemicellulose content of 13.89%, and lignin content of 14.45%. The processed SMS was stored in a dryer for subsequent experiments.

### 2.2 Isolation and screening of cellulose-degrading microbial strains

The dilution plate method was used to enrich cultivable bacteria from the SMS samples. 10 g of SMS were measured into 250 mL flasks containing 90 mL of sterile physiological saline. The mixture was shaken at room temperature and 150 r/min for 30 min. Then, 10 mL of the suspension was diluted with sterile water to three gradients: 10^−3^, 10^−4^, and 10^−5^. A total of 0.2 mL of the above gradient diluent was evenly coated in the enrichment medium and cultured at 30°C for 3 days. The enrichment medium (per liter): 10 g peptone, 3 g beef extract, 5 g sodium chloride, and 18 g agar. The color, shape, transparency, edge, and other characteristics of each colony were observed. Strains with different colony morphologies were selected for lineation separation. The purified single strain was inoculated into nutrient agar medium and stored at 4°C for subsequent experiments.

The ability of purified strains to produce enzyme activity was compared on enzyme-producing medium with carboxymethyl cellulose sodium as the sole carbon source. The strains were cultured at 30°C and 180 r/min for 3 days. The enzyme-producing liquid medium (per liter): 2 g (NH_4_)_2_SO_4_, 0.3 g MgSO_4_, 4 g KH_2_PO_4_, 0.2 g yeast extract, 3 g peptone, 10 g CMC-Na, and pH = 7.0. The fermentation broth was centrifuged at 3,000 rpm for 10 min at room temperature. The collected supernatant was the crude enzyme extract. To evaluate the enzyme activities of CMCase and FPase in the fermentation broth of different strains, we utilized the method described by Malik and Javed (2021) with slight modifications. The detailed procedure is outlined as follows.

Evaluation of FPase activity: initially, a crude enzyme solution (0.5 mL) was mixed with an acetic acid buffer solution (1.5 mL, 0.2 mM, pH = 4.8) containing 1% filter paper. The mixture was then placed in an incubator at 50°C for 1 h. The reaction was terminated with DNS reagent (2 mL). Subsequently, the mixture was then heated in a water bath at 100°C for 10 min. Assessment of CMCase activity: A crude enzyme solution (0.5 mL) was mixed with a sodium citrate buffer solution (1.5 mL, 0.2 mM, pH = 4.8) containing 0.5% CMC-Na. After incubating the mixture at 50°C for 30 min, the reaction was stopped by adding 2 mL of DNS reagent. The resulting mixture was then heated in a water bath at 100°C for 10 min. The amount of reducing sugar released in the mixture after boiling was measured at 540 nm using a microplate reader (Multiskan SkyHigh, Thermo Fisher, United States). The unit of enzyme activity was defined as the amount of enzyme needed to release 1 μmol of reducing sugar (equivalent to glucose) per minute under standard assay conditions (Rebaza et al., 2020).

### 2.3 Construction of cellulose-degrading microbial consortium

To verify whether there is antagonism among the selected efficient cellulose-degrading strains, a cross-streak method was used to inoculate the pairs of efficient cellulose-degrading strains on CMC-Na solid medium and incubated at 30°C for 3 days. The composition and components of this medium were the same as the enzyme-producing liquid medium in Section 2.2, with the addition of 1.8% agarose in 1000 mL. If the strains grow normally at the intersection of the streaks, there is no antagonism; if the intersection appears transparent without any strain growth, there is antagonism. Non-antagonistic strains were mixed in equal amounts (1:1, v/v) to form microbial consortium, which were then inoculated into enzyme-producing liquid medium and incubated at 30°C and 180 r/min for 3 days. The crude enzyme solution from each microbial consortium was collected following the method described in Section 2.2, and the CMCase and FPase activities were measured. Two microbial consortiums with higher cellulase activities were selected to evaluate their ability to produce enzymes and degrade cellulose residues.

### 2.4 Application of microbial consortium in SMS

#### 2.4.1 Production of enzymes by the microbial consortium

The production of enzymes by the microbial consortium incubated with the SMS was tested. The experimental group consisted of microbial consortiums (PX1 + F7, F16 + F) that exhibited high cellulase activity; on the other hand, the control group consisted of a microbial consortium (F7 + F8) with lower cellulase activity. The microbial consortiums were inoculated into a degradation medium with SMS as the sole carbon source. The enzyme-producing liquid medium was prepared as detailed in Section 2.2, with SMS replacing CMC-Na, while the composition and content of other substances remained unchanged. The fermentation conditions and the method for measuring enzyme activity are the same as those outlined in Section 2.2.

#### 2.4.2 Weight loss rate and composition change of SMS

The actual degradation ability of cellulose-degrading microbial. Consortium (PX1 + F7、F16 + F、F7 + F8) on SMS was evaluated by determining the total. Weight loss rate. The blank group (CK) consisted of non-inoculated strains. Except for the difference in fermentation time (20 days), the degradation medium and fermentation conditions are the same as those described in Section 2.4.1. On day 20, SMS residues were repeatedly washed three times with sterile water and dried to a constant weight in a 90°C oven. The residual mass of the SMS was weighed, and the relative degradation rate was calculated using Equations (1).


(1) $$x=\frac{M_{0}-M_{1}}{M_{0}}\times 100$$

where M_0_ represents the weight of SMS in the CK, and M_1_ represents the weight of SMS after hydrolysis by the microbial consortium.

The contents of cellulose, hemicellulose, and lignin in the residual SMS were determined by drying the cellulose residue on the 5, 10, 15, and 20th days to constant weight and using a cellulose content detection kit (Solarbio BC 4285), hemicellulose content detection kit (Solarbio BC 4445), and lignin content detection kit (Solarbio BC 4205).

### 2.5 Characterization of the SMS before and after degradation

Three groups of experiments were designed to explore the effects of a microbial consortium on SMS. The first group was the raw SMS material (RW) without any treatment. The experimental group was the SMS treated with PX1 + F7, F16 + F and the control group was the SMS treated with F7 + F8. Samples from all three groups were collected on the 5, 10, 15, and 20th days for structural characterization.

#### 2.5.1 Field emission scanning electron microscopy

The surface morphological changes in the samples were examined using FESEM (Sigma 300, Carl Zeiss, DEU). The samples were thoroughly mixed with a 2.5% glutaraldehyde solution. Then, they were placed at 4°C for 4 h. The mixture was dehydrated sequentially using ethanol solutions (v/v) of 50, 70, 80, 90, and 95% for 15 min each. Anhydrous ethanol treatment followed for a period of 20 min (Li H. et al., 2022). Next, the SMS samples were stored at −20°C for 48 h and then vacuum freeze-dried. The dried samples were sprayed with gold and then observed for morphology using FESEM at a magnification of × 2,000.

#### 2.5.2 Fourier transform infrared spectroscopy

Fourier transform infrared spectroscopy (FTIR; Nicolet iS20, Thermo Fisher Scientific, United States) was used to characterize the changes in functional groups of the SMS samples. Before analysis, the dried sample powder was treated with potassium bromide (KBr) particles. Specifically, the sample and KBr were thoroughly ground in a mortar at a ratio of 1: 100 and then pressed into pellets for 2 min at 200 kg cm^−2^. The SMS was scanned in the spectral range of 400–4,000 cm^−1^ with a resolution of 4 cm^−1^ (Liang et al., 2018).

#### 2.5.3 X-ray diffraction analysis

The crystallinity of the SMS samples was analyzed using XRD (SmartLab SE, Rigaku, Japan). The analytical conditions were as follows: the voltage applied for radiation was set at 40 kV, while the current used was 40 mA. The scanning range was 5–40 Ω, with a scanning step of 0.02 Ω (Dang et al., 2022). Refer to Equations (2) to calculate the relative crystallinity using the Segal’s method (Massironi et al., 2022).


(2) $$CrI=\frac{I_{002}-I_{\mathrm{am}}}{I_{002}}$$

where CrI represents the relative crystallinity of the sample; *I*_002_ represents the maximum diffraction peak intensity in the crystalline region at approximately 2θ = 22°; and *I*_am_ represents the diffraction peak intensity in the amorphous region at approximately 2θ = 18°.

### 2.6 Identification of cellulose-degrading bacteria

The bacterial genomic DNA extraction kit (Beijing Solabao Technology Co., Ltd.) was used to extract DNA from cellulose-degrading bacteria. The universal primers 27F (5’-AGAGTTTGATCCTGGCTCA-3′) and 1492R (5’-TACGGCTACCTTGTTACGACTT-3′) were used to amplify partial sequences of 16S rRNA. The gyrA gene was amplified with specific primers gyrA-F (5′-CAGTCAGGAAATGCGTACGTCCTT-3′) and gyrA-R (5′-CAAGGTAATGCTCCAGGCATTGCT-3′). PCR of the 16S rRNA gene fragment (95°C for 5 min; 30 cycles, 95°C for 60 s, 55°C for 60 s, 72°C for 1 min, and finally 10 min at 72°C). PCR of the gyrA gene fragment (95°C for 3 min; 32 cycles, 95°C for 30 s, 58°C for 30 s, 72°C for 1 min, and finally 10 min at 72°C). The PCR productions were sent to Shanghai Shenggong Biotechnology Co., Ltd. The obtained sequences were compared for homology with the sequences in the NCBI database^1^ using BLAST. The phylogenetic tree was constructed using MEGA 7.0^2^ to determine the species relationships of cellulose-degrading bacteria.

### 2.7 Data analysis

The data were processed using software such as Origin, Mega 7.0, GraphPad Prism, and SPSS 6.0. All experiments were repeated at least three times, and the average ± standard deviation denotes the statistical significance of each data point. The statistical confidence interval was set at 95%, and statistical significance was considered valid when *p* < 0.05.

## 3 Results

### 3.1 Screening of cellulose-degrading bacteria

**Figure 1:**
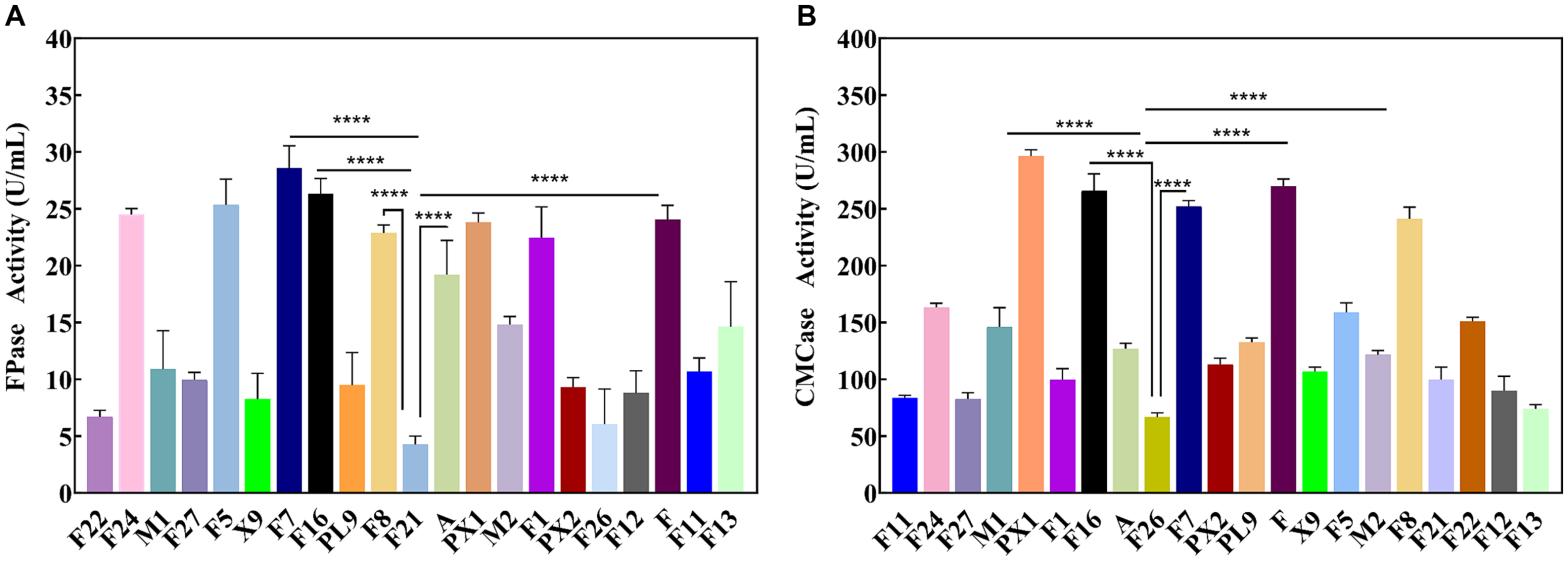
Enzyme activity of cellulose degrading strain. **(A)** FPase activity. **(B)** CMCase activity. ^****^p value < 0.0001.

Through enrichment culture, 49 different morphological characteristics of bacteria were obtained from the SMS samples. Enzyme activity tests revealed that 21 strains exhibited cellulase activity. The results of the FPase and CMCase enzyme activities are shown in Figure 1. Strain F7 exhibited the highest FPase activity, reaching 28.57 U/mL, which was 21.88 U/mL higher than the lowest enzyme activity observed in strain F21. It was also found that except for strain F7, strains PX1, F, F16, and F8 had FPase activities greater than 22.00 U/mL, which were 23.81, 24.04, 26.32, and 22.88 U/mL, respectively (Figure 1A). Strain PX1 had the highest CMCase activity, reaching 296.30 U/mL. The CMCase activities of strains F, F16, F7, and F8 were also higher than 240.00 U/mL, which were 269.99, 265.79, 252.31, and 241.12 U/mL, respectively (Figure 1B). Relevant studies have shown that the level of cellulase activity reflects the strength of the cellulose degradation ability of strains (Yi et al., 2022). Therefore, in this study, strains PX1, F, F16, F7, and F8 were selected as the target strains for constructing a cellulose-degrading microbial consortium.

### 3.2 Construction of cellulose-degrading microbial consortium

**Figure 2:**
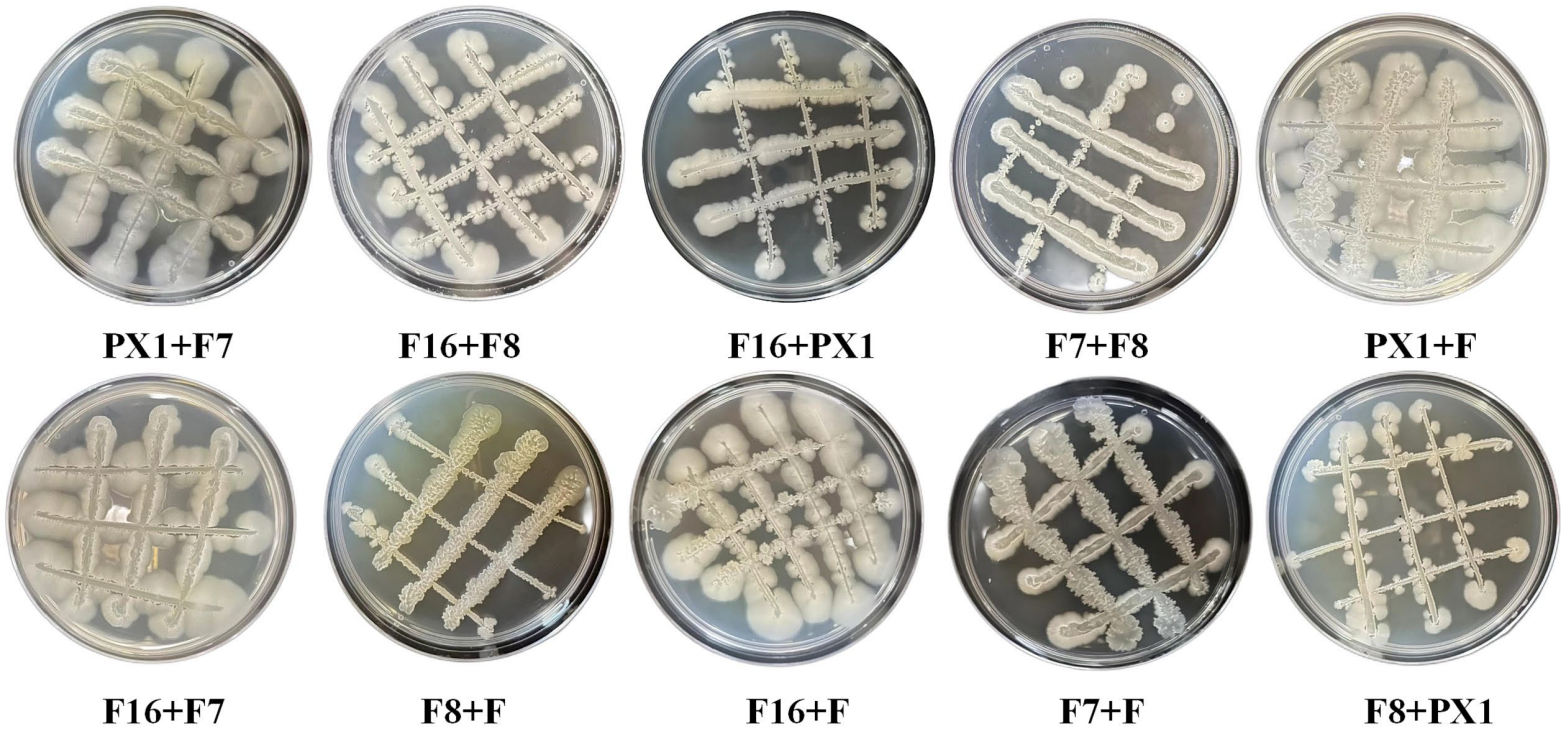
Antagonism results of cellulose-degrading microbial consortium.

**Figure 3:**
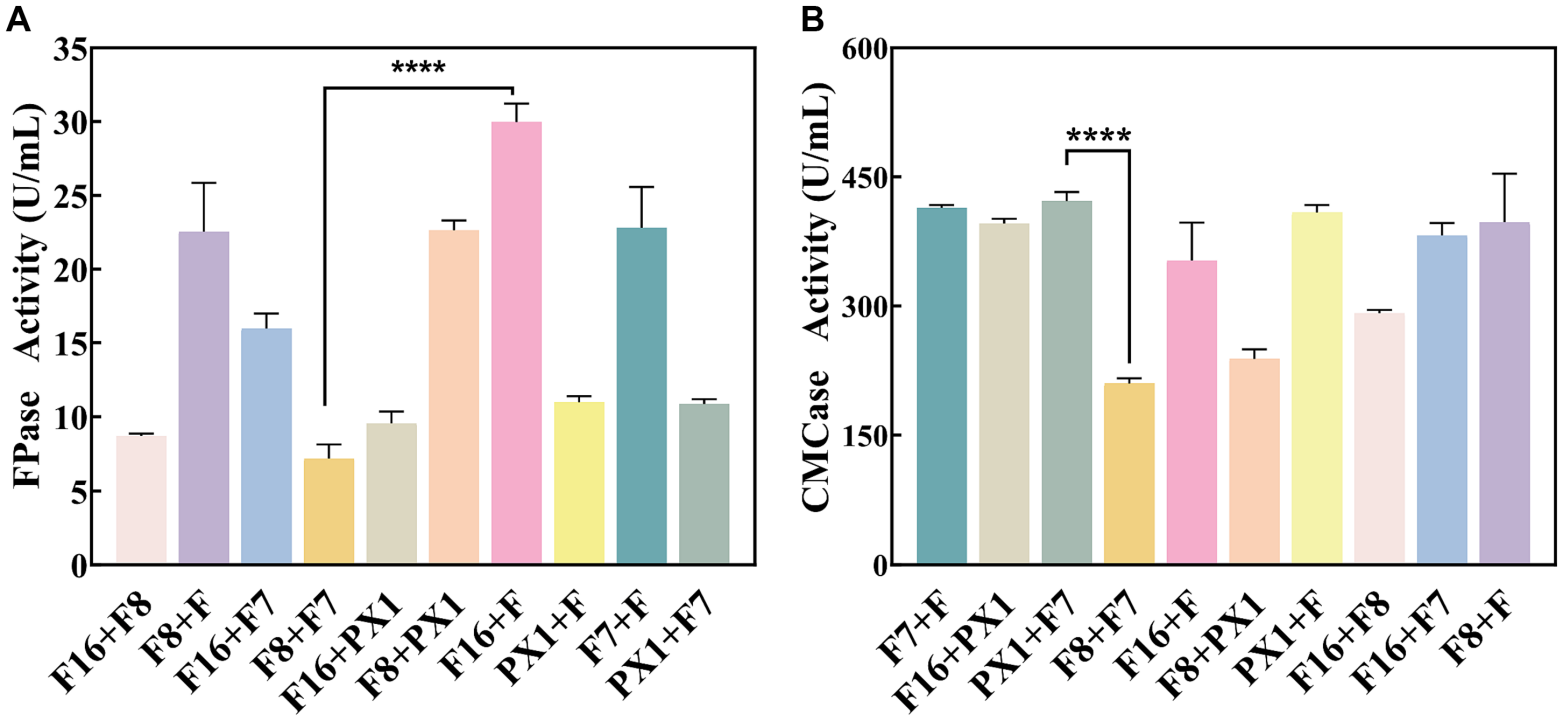
Enzymatic activity of cellulose-degrading microbial consortium. **(A)** FPase activity. **(B)** CMCase activity. ^****^p value < 0.0001.

The study found that there was no antagonism between the target strains PX1, F, F16, F7, and F8 (Figure 2). Therefore, it was chosen to combine the two strains to construct 10 groups of cellulose-degrading microbial consortium. There were differences in enzyme activity among these microbial consortiums (Figure 3). Comparing the FPase activity of the cellulose-degrading microbial consortium with that of the single strains in Section 3.1, it was found that the FPase enzyme activity of F16 + F was the highest, at 29.96 U/mL. This value was higher than the enzyme activity of any single strain in the corresponding combination, 12.17% higher than that of strain F16, and 19.78% higher than that of strain F. However, the FPase enzyme activity of the other microbial consortiums was lower than the minimum enzyme activity of the corresponding single strain in the combination. For example, the FPase enzyme activity of F7 + F8 was 7.20 U/mL, significantly lower than the enzyme activity of strain F8 (22.88 U/mL). Similarly, the CMCase activity of the microbial consortium F8 + PX1 and F7 + F8 was also lower than that of the corresponding single strains. Fortunately, there were eight groups of microbial consortium whose CMCase activity was higher than that of any single strain in the corresponding combination. For example, the CMCase activity of PX1 + F7 reached 421.94 U/mL, which was 42.41% higher than that of strain PX1 and 67.23% higher than that of strain F7. Microbial consortium PX1 + F exhibited a CMCase activity of 408.69 U/mL, which was 41.39% higher than that of strain F. Another example was the CMCase enzyme activity of F7 + F, which was 413.92 U/mL, 53.32% higher than that of strain F. The results showed that F16 + F exhibited the highest FPase activity, PX1 + F7 exhibited the highest CMCase activity, and F7 + F8 exhibited the lowest FPase and CMCase activity. Therefore, we selected these three microbial consortiums for subsequent experimentation.

### 3.3 Analysis of the enzyme production capacity and degradation performance of cellulose-degrading microbial consortium on SMS

#### 3.3.1 Production of enzymes by the microbial consortium

**Figure 4:**
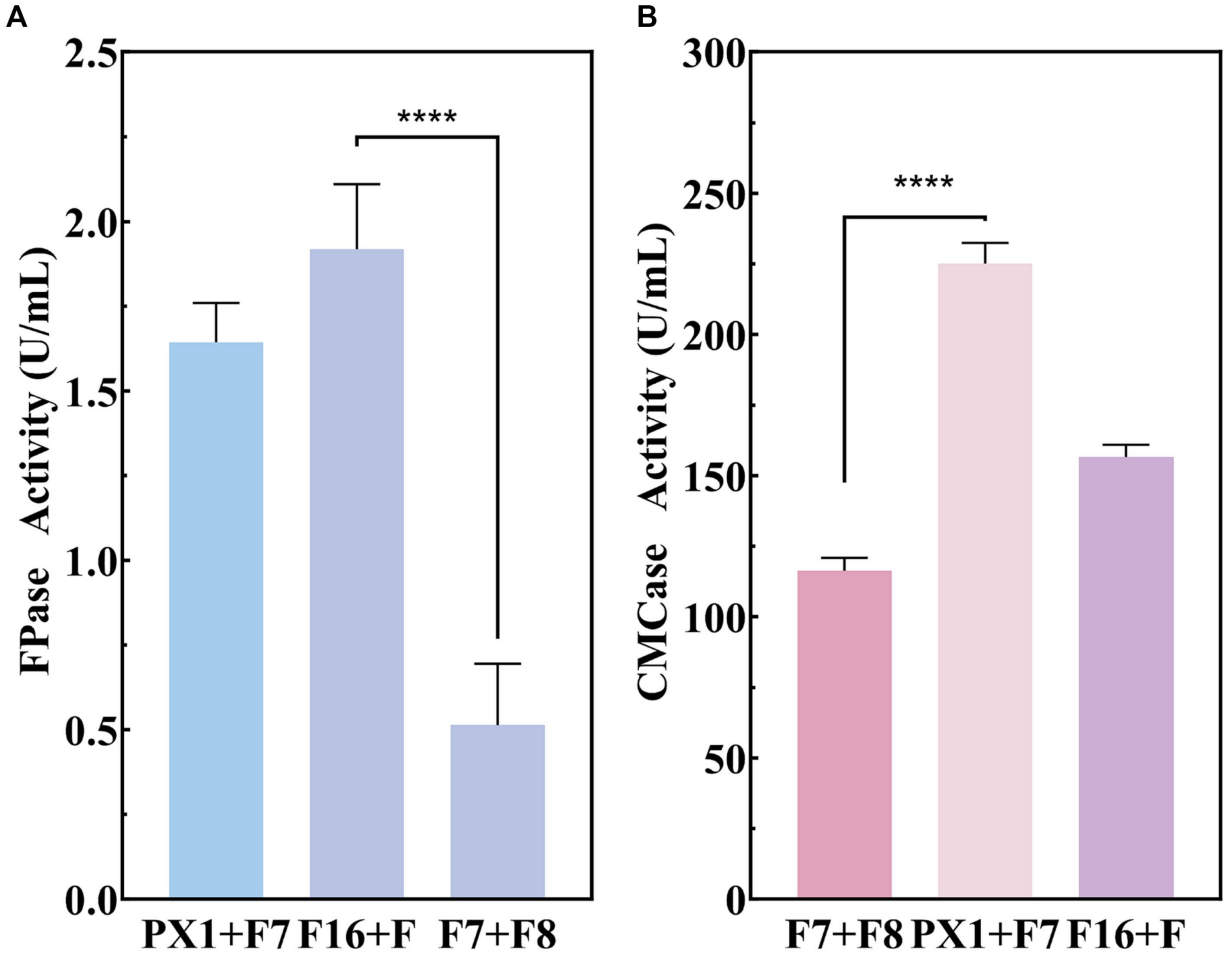
Enzymatic activity of cellulose-degrading microbial consortium in SMS. **(A)** FPase activity. **(B)** CMCase activity. ^****^p value < 0.0001.

The production of enzyme was carried out to evaluate the cellulase production capacity of the microbial consortium with SMS as the sole carbon source. As shown in Figure 4, F16 + F and PX1 + F7 with corresponding CMCase activities are 225.16 and 156.63 U/mL, while the FPase activities are 1.91 and 1.64 U/mL, respectively. The FPase activity of F7 + F8 was 0.51 U/mL, and the enzyme activity of CMCase was 167.88 U/mL. The above experimental results indicated that the constructed microbial consortium could effectively utilize SMS as the sole carbon source and secrete cellulase significantly.

#### 3.3.2 Analysis of the weight loss rate and composition changes of SMS

**Figure 5:**
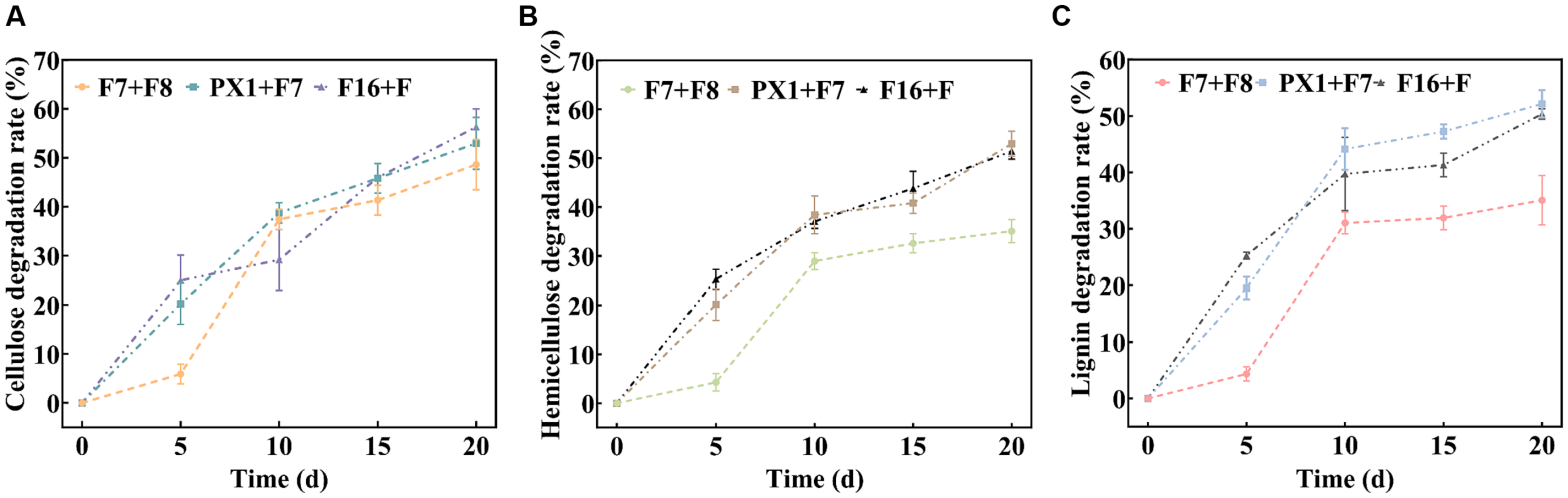
Analysis of the lignocellulose degradation process. **(A)** Cellulose degradation rate. **(B)** Hemicellulose degradation rate. **(C)** Lignin degradation rate.

The results of the SMS total weight loss rate are shown in Table 1. After 20 days of fermentation, PX1 + F7 had the highest weight loss rate of SMS, reaching 26.08%, which was 13.30% higher than that of F7 + F8. The study found that the degradation rates of lignin, cellulose, and hemicellulose in SMS by cellulose-degrading microbial consortium increased with time during fermentation (Figure 5). Among them, PX1 + F7 had the highest degradation rates of hemicellulose and lignin in SMS, which were 52.96 and 52.13%, respectively. F16 + F exhibited the strongest cellulose degradation ability in SMS, reaching 56.30%. In contrast, F7 + F8 exhibited relatively lower degradation rates of hemicellulose, cellulose, and lignin in SMS, which were 35.04, 35.05, and 48.62%, respectively. The results indicate that the degradation of lignin, cellulose, and hemicellulose by microbial consortium is a continuous process. Furthermore, microbial consortium with higher cellulase activity exhibit more effective degradation of SMS.

**Table 1:** The total weight loss rate of residue after 20 days of hydrolysis.

Samples	CK	F7 + F8	F16 + F	PX1 + F7
Initial amount of residue (g)	0.5	0.5	0.5	0.5
Residual amount after 20 days of fermentation (g)	0.4676	0.4078 ± 0.0075	0.3647 ± 0.0016	0.3456 ± 0.0032
Weight-loss ratio (%)	6.48	12.78	22.00	26.08

### 3.4 Effect of microbial consortium on the structural properties of SMS

#### 3.4.1 Scanning electron microscopy analysis of the surface microstructure of SMS

**Figure 6:**
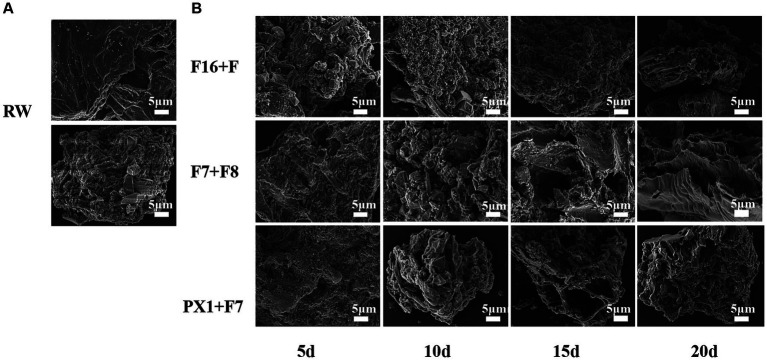
Scanning electron microscope image of the samples. **(A)** Raw materials for SMS (RW). **(B)** SMS treated by different cellulose-degrading microbial consortium.

The FESEM results of the raw SMS material (RW) and the SMS samples treated with microbial consortium are shown in Figure 6. The study found that the surface morphology of SMS in RW exhibited a smooth and compact surface with better integrity (Figure 6A). However, after 20 days of degradation treatment by different microbial consortium, the surface microstructure of SMS in PX1 + F7, F16 + F, and F7 + F8 showed different changes (Figure 6B). On the 5th day, many microorganisms were observed on the surface of SMS samples. PX1 + F7 specifically exhibited the highest number of bacterial colonies. On the 10th day, the surface of the SMS samples showed irregular and rough structures. In particular, F16 + F showed an increased number of bacterial colonization on the surface of SMS, while PX1 + F7 exhibited a decreased number of bacterial cells on the surface of SMS, and the outer thin-walled tissue gradually disappeared, exposing the inner thin-walled tissue, corresponding to a fuzzy appearance on the surface of SMS. On the 15th day, the number of bacterial colonization on the surface of SMS gradually decreased in PX1 + F7 and F16 + F. With the passage of fermentation time, the surface structure of SMS underwent significant changes, with the outer thin-walled tissue almost completely degraded and peeled off, accompanied by uneven cracks and gaps. In contrast, there was no significant change in the surface of the SMS in the control group F7 + F8 compared to 10 days earlier. On the 20th day, the fiber density of the SMS samples in the experimental groups PX1 + F7 and F16 + F was lost, showing a loose and porous honeycomb-like tissue structure, while the inner thin-walled tissue of the surface of SMS in F7 + F8 was gradually exposed, resulting in an irregular rough surface structure.

#### 3.4.2 Fourier transform infrared spectroscopy

**Figure 7:**
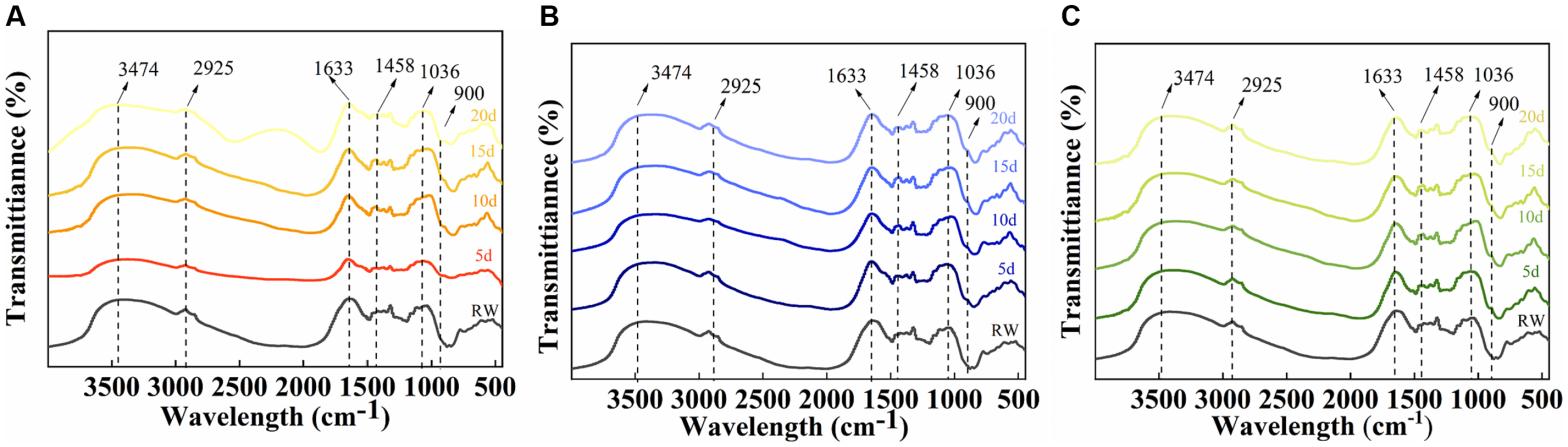
Infrared spectral analysis of the raw material for SMS (RW) and the SMS treated with different cellulose-degrading microbial consortium (5–20 days). **(A)** F16 + F; **(B)** PX1 + F7; and **(C)** F7 + F8.

To investigate the degradation mechanism of SMS by a cellulose-degrading microbial consortium, we analyzed the functional groups in the fiber structure of the raw SMS materials and the SMS treated with cellulose-degrading microbial consortium using FTIR. The study found that the typical peaks of cellulose, hemicellulose, and lignin in the SMS samples changed little after degradation by the microbial consortium (Figure 7). Compared with RW, the absorption peaks of F16 + F, PX1 + F7, and F7 + F8 at 3,474 and 2,925 cm^−1^ shifted to lower wavenumbers, representing the tensile vibration of O-H in the hydroxyl group in the fiber structure and the tensile vibration of C-H in methyl and methylene, respectively. The absorption peaks of the three groups at 1,633 cm^−1^ also shifted to a lower wavenumber, which was related to the aromatic carbonyl vibration of lignin (Dong et al., 2019; Huang et al., 2021). Similarly, the absorption peaks at 1,458 cm^−1^ in the three groups shifted to a lower wavenumber, and a new absorption peak appeared at 900 cm^−1^. The absorption peaks at 1,458 cm and 900 cm^−1^ are associated with a mixture of cellulose I, cellulose II, and amorphous cellulose (Liu et al., 2015). In addition, it was found that the absorption peaks of the three groups of samples all appeared at 1,036 cm^–1^, and the absorption peaks of F16 + F and PX1 + F7 shifted to higher wavenumbers, and the absorption peaks at this position were related to the tensile vibration of C-O (Dong et al., 2019).

#### 3.4.3 X-ray diffraction

**Figure 8:**
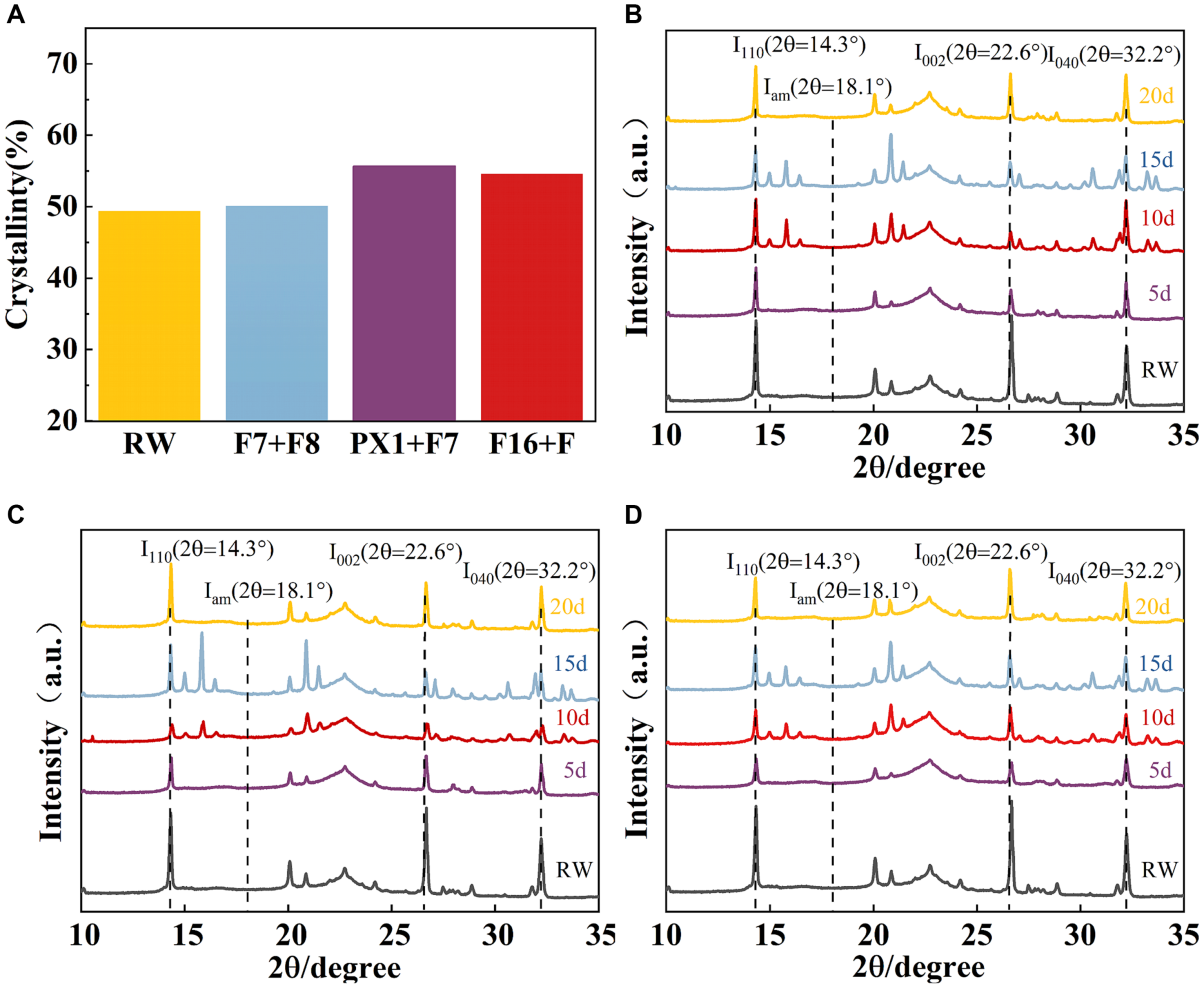
**(A)** The results of crystallinity and X-ray diffraction. Crystallinity of SMS for three groups of microbial consortium at different processing times. **(B)** PX1 + F7; **(C)** F7 + F8; and **(D)** F16 + F.

The crystal structure of the raw materials for SMS (RW) and the SMS treated with different cellulose-degrading microbial consortiums was analyzed using an X-ray diffractometer. Compared to the RW, the experimental groups F16 + F, PX1 + F7, and the control group F7 + F8 showed an increase in CrI values from 49.36 to 50.11, 55.68, and 54.59%, respectively (Figure 8A). The study results indicate that all samples showed three distinct diffraction peaks near 2θ = 14.3, 22.6, and 32.2°, corresponding to the 110, 002, and 040 crystal planes of cellulose, respectively, which is a typical cellulose I structure (Jiao et al., 2019) (Figures 8B–D). In the three groups of samples, the XRD diffraction peak positions did not change significantly, indicating that the microbial consortium cannot cause a significant change in the cellulose crystal structure of the SMS (Luo et al., 2018). However, the diffraction peak intensities at 2θ = 14.3, 22.6, and 32.2° decreased compared to RW, indicating a change in SMS crystallinity after treatment with the microbial consortium.

### 3.5 Identification results of cellulose-degrading bacteria

**Figure 9:**
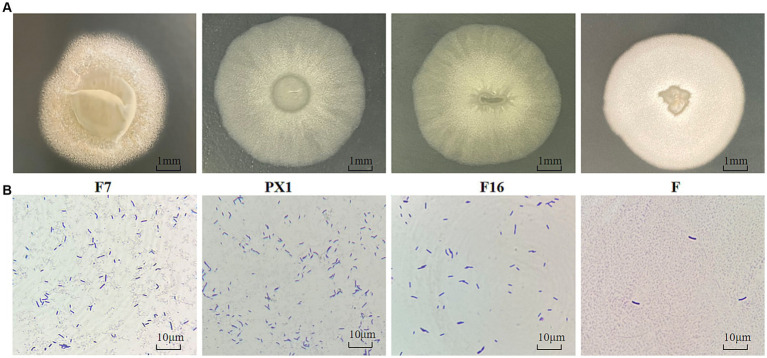
Identification of cellulose-degrading bacteria. **(A)** Colony morphology diagram. **(B)** Diagram of single colony morphology.

The colonies of strain F7 were irregularly round, pale yellow, and opaque, with irregular edges and a rough surface with folds and a raised center. The colonies of strain F were round, milky white, and opaque, with a folded surface and a central depression resembling a volcanic crater. The colonies of strain PX1 were round, white, semitransparent, and flat, with a central depression resembling a volcanic crater. The colonies of strain F16 were irregularly round, white, semitransparent, and flat and had a folded surface (Figure 9A). Under the optical microscope, all four strains had individual cells that were elongated rods with blunt ends (Figure 9B).

**Figure 10:**
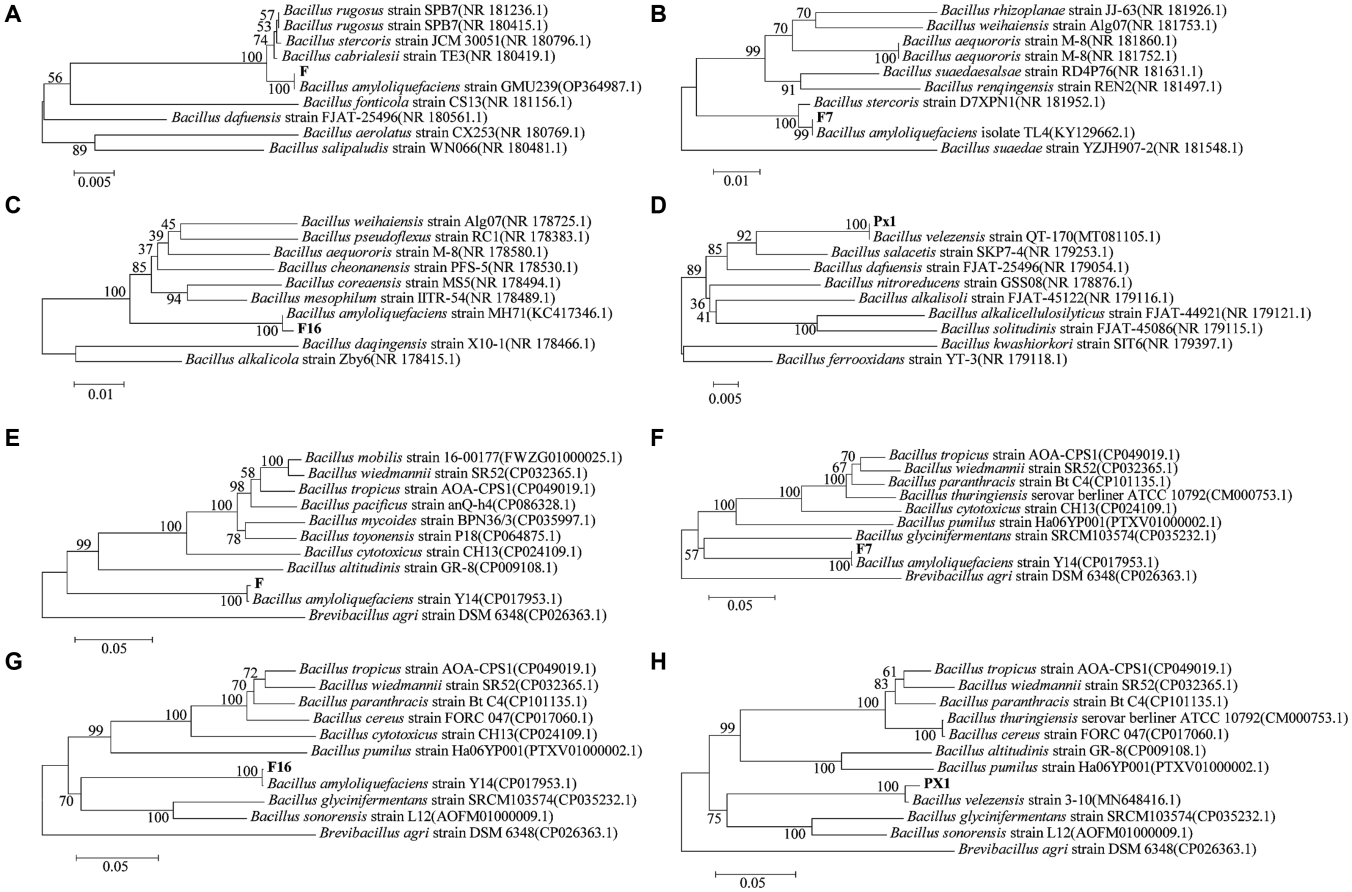
Phylogenetic trees generated based on sequences of 16S rRNA **(A–D)** and gyrA **(E–H)** regions of cellulose-degrading bacteria.

The 16S rRNA sequences of strains PX1, F7, F, and F16 were related to *Bacillus amyloliquefaciens* and *Bacillus velezensis* by BLAST alignment analysis in the NCBI database, with 99% sequence homology (Figures 10A–D). However, similar relatives of *Bacillus* such as *B. amyloliqueciate* and *B. velezensis*. It has similar phenotypic properties and extremely high sequence homology of the 16S RNA gene. In order to further determine the taxonomic status of the strains, the gyrA genes of the four strains were analyzed sequentially. The sequence similarity between strain F7 and *B. amyloliqueciate* (CP012953.1) was 100%, the sequence similarity between strain F16 and *B. amyloliqueciate* (CP072311.1) was 100%, and the sequence similarity between strain F and *B. amyloliqueciate* (CP017953.1) was 100%. The phylogenetic tree constructed by the NJ method showed that these three strains were clustered in the same branch as *B. amyloliqueciate*, indicating that the three strains belong to the same genus *Bacillus*. Strain PX1 was in the same clade as *B. velezensis* (MN648416.1), and the sequence similarity was 100%, indicating that the strain PX1 was *B. amyloliqueciate* (Figures 10E–H).

## 4 Discussion

### 4.1 Enzyme-producing capacity of single strains and microbial consortium

Spent mushroom substrate is one of the important lignocellulosic resources. However, due to the complex structure and stable chemical properties of lignocellulose, which is a highly oxidized polymer (Mackenzie and Francis, 2013). Cellulose-degrading microorganisms, which have the ability to produce a variety of related enzymes, play an important role in the utilization of waste biomass resources. Therefore, it is of great significance to screen cellulose-degrading bacteria from the environment in order to promote the bioconversion of SMS and increase its utilization rate. The study screened 21 strains of cellulose-degrading bacteria, among which *B. amyloliquefaciens* (F16, F, F7) and *B. velezensis* (PX1) exhibited higher enzyme activity. Previous reports have confirmed that *B. amyloliquefaciens* and *B. velezensis* both exhibit strong cellulose degradation activity (Hussain et al., 2017; Cheng M. et al., 2022). Li J. et al. (2022) isolated a cellulase-producing strain, *B. amyloliquefaciens* DC-2, with an FPase activity of 7.31 U/mL, while the cellulose-degrading strains screened in this study exhibited high FPase activities ranging from 23.81 to 28.57 U/mL, indicating greater enzyme activity compared to strain DC-2. A cellulase-producing strain, *Bacillus subtilis* K1, was screened from crop straw, with CMCase activity of 24.69 U/mL and β-Gase activity of 1.14 U/mL (He et al., 2023). From pig manure, a strain of *B. velezensis* M2 was screened, with CMCase activity of 61.50 U/mL under optimal conditions (Li D. et al., 2020). The CMCase activities of the cellulose-degrading strains screened in this study were higher than 240.00 U/mL, indicating higher cellulase production characteristics compared to the findings of the aforementioned scholars.

In natural environments, the degradation of lignocellulosic fibers is frequently the result of the participation of multiple microorganisms. Cultivating a combination of strains with complementary enzyme systems can not only compensate for the incomplete enzyme systems produced by some strains or the low enzyme activity of individual strain, but also slow down the feedback inhibition, thereby improving the efficiency of cellulose degradation (Wei et al., 2019). This study found through enzyme activity experiments that the CMCase activity of PX1 + F7 was 42.41% higher than that of strain PX1 and 67.23% higher than that of strain F7. This conclusion is consistent with the results of Liu et al. (2022), whose study indicated that the microbial consortium constructed by *Streptomyces* CPA-3-4, *Micromonospora* GS-3-39, and *Streptomyces* GS-4-21 significantly increased the activities of FPase, C1, Cex, and β-Gase by 102.01, 96.81, 30, and 59.15%, respectively. Zhang and Dong (2022) used seven different cellulose-degrading bacteria to form a microbial consortium VI. The CMCase, FPase, and β-Gase activities of this consortium were 141.89, 104.56, and 131.18 U/mL, respectively. The enzyme activities mentioned above were increased by 364, 265, and 296% compared to the highest enzyme activities of strain A-2. In addition, the research also found that applying the microbial consortium VI to compost not only improved the efficiency of composting but also significantly increased the nutrient content of the compost. Consequently, some scholars have proposed that the degradation of cellulose or lignin-like substances, the synergistic effects produced by the interaction of microbial consortium are more advantageous than those of single strains (Chen et al., 2019). Notably, this study also found that the enzyme activity in the fermentation liquid of the microbial consortium PX1 + F8 and F7 + F8 was lower than that of the single strains, which is consistent with the results of Cheng Y. et al. (2022). The CMCase activity of the microbial consortium (N13 + N21) was also lower than that of the single strains. In the microbial consortium MCHCA, constructed by Poszytek et al. (2016), some strains did not enhance the cellulase activity of the MCHCA strain, but instead reduced the overall enzyme activity. The reason for this phenomenon may be the secretion of competitive inhibitors among non-antagonistic strains, which leads to a decrease in cellulase activity in microbial consortium (Wei et al., 2019).

In the study, it was found that the enzyme activity in CMC-Na medium was higher than that in SMS medium. CMC-Na can better induce cellulase production in extracellular cells than other natural substrates (Sajith et al., 2016; Malik and Javed, 2021; Da Silva et al., 2022). In CMC-Na medium, the CMCase activity of PX1 + F7 was 421.94 U/mL, the activity of F16 + F was 29.96 U/mL. However, in SMS medium, the microbial consortium produced less cellulase activity, and the enzyme activity of PX1 + F7 CMCase was 225.16 U/mL, the activity of FPase enzyme was 14.57 U/mL for F16 + F. These results support the idea that natural carbon sources can induce cellulase production, albeit to a lower extent than CMC-Na.

### 4.2 SMS composition changes during microbial consortium degradation

The microbial consortium F16 + F, which was constructed in this study, exhibits a strong ability to degrade cellulose, with a degradation rate of 56.30%. Moreover, the microbial consortium PX1 + F7 exhibits a strong ability to degrade lignin and hemicellulose, with degradation rates of 52.13 and 52.96%, respectively. The degradation ability of the microbial consortium constructed in this study for SMS was close to that of other lignin-containing substances. In a study conducted by Pardeep and Kocher (2018), a microbial consortium constructed with *Pleurotus ostreatus* and *Phanerochaete chrysosporium* achieved degradation rates of 43.0% for cellulose, 12.7% for hemicellulose, and 7.0% for lignin in straw under optimal conditions. In another study by Chu et al. (2020), a microbial consortium CB was constructed with *P. chrysosporium*, *Trametes versicolor*, and *P. ostreatus* achieved degradation rates of 43.36% for lignin, 31.29% for cellulose, and 48.36% for hemicellulose in fermented straw after 20 days. Compared to the above studies, the cellulose microbial consortiums constructed in this study exhibit outstanding degradation ability during the SMS degradation process.

### 4.3 Structural characterization of SMS after degradation

The biodegradation process mainly relies on the action of enzymes attached to the cell surface, which causes the material surface to undergo degradation (Joshi et al., 2022). Compared to the RW, the surface of the SMS sample treated with cellulose-degrading microbial consortium clearly shows bacterial colonization, as well as the cracks and porous structure formed by them. Similar to the results of this study, Corrêa et al. (2016) also found that the surface of the SMS treated with *Pleurotus pulmonarius* exhibited varying degrees of small pores and cracks. The changes in the SMS structure are the result of the interaction between the microbial consortium and the SMS surface, providing effective evidence of lignocellulosic biodegradation (Arumugam et al., 2018). Experimental groups with higher enzyme activity, F16 + F and PX1 + F7, showed faster and more significant changes in the surface structure of SMS compared to F7 + F8, which had lower enzyme activity. This phenomenon indicates that cellulase plays a crucial role in the enzymatic hydrolysis of lignocellulosic materials.

According to the FIR spectrum, the blank group of RW and the SMS samples treated with microbial consortium had the same structure of functional groups. This indicates that the addition of the microbial consortium did not alter the functional group types of SMS. The difference lies in the change in absorption peak intensity after treatment with a microbial consortium. The study by Indumathi et al. (2022) found similar changes in the FTIR spectra of pulp after deinking treatment with cellulase, mainly attributed to the disruption of cellulose structure by the microbial consortium. Notably, the addition of the microbial consortium resulted in enhanced absorption peaks at 2,925 cm^−1^ and the emergence of new absorption peaks at 900 cm^−1^, both of which validate the occurrence of the cellulose hydrolysis process (Dar et al., 2021). These changes indicate that the microbial consortium can utilize SMS as a carbon source to produce metabolic products, such as reducing sugars, which promote the cocultivation of strains by providing nutrients.

Additionally, in the XRD analysis results, the characteristic diffraction peak positions of F16 + F, PX1 + F7, and F7 + F8 are essentially identical to those of the RW, but with different intensities. This suggests that the ordered structure of the crystalline region in cellulose remained intact despite the colonization of the microbial consortium and enzyme contact (Zhang et al., 2021). In the three groups of samples, the XRD diffraction peak positions did not change significantly, indicating that the microbial consortium cannot cause a significant change in the cellulose crystal structure of the SMS (Luo et al., 2018). However, compared to the RW, the diffraction peak intensities at 2θ = 14.3, 22.6, and 32.2° weakened, indicating a change in crystallinity, possibly related to the destruction of the 110, 002, and 004 crystal planes during the enzymatic hydrolysis process (Chen et al., 2019). Research has found that the CrI values of the three sample groups are higher than those of the RW. The increase in CrI values was mainly due to the removal of amorphous lignin and hemicellulose from the cell wall, which increased proportions of crystalline cellulose (Rajput and Zeshan Visvanathan, 2018; Li F. et al., 2020). Compared to crystalline cellulose, amorphous lignin and hemicellulose in the cell wall are more susceptible to attack by microorganisms and enzymes. This leads to an increase in the surface porosity of the cell wall structure, resulting in extensive degradation. Therefore, the crystallinity of cellulose molecules has a significant impact on the rates of enzymatic hydrolysis. Yan et al. (2021) found that the CrI value of corn stover increased after treatment, as the degradation of amorphous biomass, including hemicellulose, which exposed more binding sites. Our research results are also consistent with those of Fu et al. (2022), who found that the crystallinity index of coconut oil residue increased after treatment with *M. guillermondii*.

In this study, we screened cellulose-degrading bacteria with a strong ability to degrade cellulose, and investigated the degradation performance of SMS using microbial consortium. In the future, it is necessary to further carry out practical production and application research, such as strain ratio, microbial agent formulation optimization, fermentation condition optimization, etc., to improve the degradation efficiency of SMS. At the same time, each strain within the microbial consortium possesses its own unique characteristics and functions. The study of how different strains within a microbial consortium respond to the environment through signal molecule transmission and carry out division of labor to accomplish different tasks holds significant research value in the field of molecular biology. Therefore, exploring the mutual cooperation among cellulose-degrading microbial consortium is an important measure to enhance the efficiency of utilizing lignocellulosic resources. It is also one of the directions for our future research.

## 5 Conclusion

Four cellulose-degrading strains were successfully screened from the SMS and identified as *B. amyloliquefaciens* (F16, F, and F7) and *B. velezensis* (PX1), based on sequences of their 16S rRNA, gyrA gene. The structure of the raw SMS material and the SMS treated with the microbial consortium was characterized by SEM, FTIR, XRD, and other techniques. The actual degradation ability of the microbial consortium for SMS was evaluated, and the potential for biotransformation of SMS by the microbial consortium was revealed. This study confirmed the efficiency of degrading SMS through the construction of high-yield cellulase microbial consortium. The research results are of great significance for the degradation of biomass from agricultural waste.

## Data availability statement

The datasets presented in this study can be found in online repositories. The names of the repository/repositories and accession number(s) can be found below: https://www.ncbi.nlm.nih.gov/nuccore, PP400934, PP400935, PP400936, and PP400978.

## Author contributions

JL: Methodology, Investigation, Data curation, Conceptualization, Writing – review & editing, Writing – original draft. XW: Supervision, Methodology, Investigation, Data curation, Conceptualization, Writing – review & editing, Writing – original draft. SQ: Resources, Project administration, Methodology, Investigation, Funding acquisition, Formal analysis, Data curation, Conceptualization, Writing – review & editing. WZ: Supervision, Methodology, Investigation, Data curation, Writing – review & editing, Project administration. SZ: Resources, Investigation, Writing – review & editing, Funding acquisition. KS: Investigation, Writing – review & editing, Project administration. LX: Methodology, Investigation, Writing – review & editing, Project administration. XM: Writing – review & editing, Project administration. XZ: Formal analysis, Data curation, Writing – review & editing.

## Footnotes

^1^ https://www.ncbi.nlm.nih.gov/

^2^ https://www.megasoftware.net
